# Vascular functional tests and preemptive medicine

**DOI:** 10.1038/s41440-020-00546-5

**Published:** 2020-09-03

**Authors:** Atsushi Tanaka, Shigeru Toyoda, Koichi Node

**Affiliations:** 1 Department of Cardiovascular Medicine, Saga University, Saga, Japan; 2 Department of Cardiovascular Medicine, Dokkyo Medical University, Mibu, Tochigi Japan

**Keywords:** Vascular function, Arterial stiffness, Pressure wave reflection, Cardiovascular risk factors, Preemptive medicine

In 2016, the Japan Stroke Society and the Japanese Circulation Society jointly released their “5 Year Plan for Overcoming Stroke and Cardiovascular Disease” [1]. To solve the underlying problems in cardiovascular medicine and achieve the goal of improving prognosis, five strategies were proposed: development of human resources, improvement of the medical system, promotion of registry operations, prevention of disease and enhancement of public awareness, and strengthening of clinical and basic research. In the section on prevention and public awareness, the establishment of a data bank for the promotion and spread of preemptive medicine utilizing biomarkers and vascular function tests was proposed. After receiving that proposal, the Japan Society for Vascular Failure proposed physiological diagnostic criteria for several vascular functional tests, including pulse wave velocity (PWV), to identify possible subjects with vascular failure [2, 3], although the augmentation index (AI) was not included in that proposal. The important next step was to collect real-world data from various types of populations and to analyze the association with clinical relevance. There is also a need for further evidence on the effect of postintervention changes in vascular function on future outcomes.

In this issue of the journal, Fujii et al [4]. investigated differences in the longitudinal associations of conventional cardiovascular risk factors with arterial stiffness for macrovascular damage and with pressure wave reflection for microvascular damage using data obtained over 9 years (median 7 years) in middle-aged Japanese working men. In this analysis, they used a mixed-model linear regression analysis for repeated-measures data to exclude the confounding effects of time-varying variables, resulting in a reliable estimation of the longitudinal associations of interest. Our first impression of their findings was that each of the conventional cardiovascular risk factors had more heterogeneous associations than expected with the changes in arterial stiffness and pressure wave reflection. This may be in part quite reasonable when using real-world data obtained from a heterogeneous population in a metropolitan area such as Tokyo. However, we can learn several important findings and clinical implications from the study by Fujii et al. [4].

First, this is the first study that assessed the longitudinal associations of conventional cardiovascular risk factors with arterial stiffness and pressure wave reflection in a working male population. As expected, both the brachial-ankle PWV and radial AI increased annually with aging. Interestingly, in their cohort, the percentages of patients receiving medications for comorbidities, such as hypertension and dyslipidemia, also apparently increased during the follow-up interval, possibly affecting their study results. Because those medications often modify some of the clinical parameters examined, including high-density lipoprotein cholesterol, it should be clinically valuable to examine the effect of such therapeutic interventions on vascular function. Although we cannot overcome aging completely, we will be able to take steps to address it by making appropriate lifestyle modifications and taking medications.

Second, their study clearly demonstrated that each blood pressure (BP) variable, including systolic, diastolic, and mean BP, was independently and longitudinally associated with brachial-ankle PWV and radial AI, highlighting the importance of longitudinal BP management to attenuate macro- and microvascular damage. An important aim of performing vascular function tests in hypertensive patients is to noninvasively ascertain information on arterial damage and dysfunction at an asymptomatic stage to improve the prevention and treatment of cardiovascular disease. Accumulated evidence suggests that BP-lowering therapy could improve vascular function; however, it is also known that such effects differ among classes of antihypertensive agents. Further study is therefore needed to determine the appropriate use of antihypertensive agents and vascular functional tests according to individual pathophysiological conditions. In a statement of the plan for the future by the Japanese Society of Hypertension [5], it was advocated to promote “Future Medicine”, which utilizes artificial intelligence, big data, and the Internet of things to enable the next generation of the prevention, prediction, and control of hypertension. Thus, it will be increasingly necessary to incorporate big data on vascular functional tests into the care of hypertension.

Third, abnormal glucose metabolism was surprisingly not longitudinally associated with augmented wave reflection representing microvascular damage. As noted by the authors, this was partly due to the measurement principle of radial AI. In patients with abnormal glucose metabolism and diabetes, microvascular complications often reduce the patients’ quality of life and worsen their prognosis, suggesting an urgent need for the early and precise detection of microvascular dysfunction. Therefore, it would be highly warranted to verify whether (and if so, how) abnormal glucose metabolism affects microvascular function by use of other vascular functional tests and biomarkers.

The longitudinal effects of lipid metabolism were also inconsistent between macro- and microvascular function. A systematic review and meta-analysis of randomized controlled trials demonstrated that statin therapy reduced aortic PWV [6], while in a representative cohort of the Anglo-Scandinavian Cardiac Outcomes Trial (ASCOT), statin therapy in patients in the Conduit Artery Function Evaluation-Lipid-Lowering Arm (CAFE-LLA) did not affect central BP and AI [7]. Thus, the relationship between dyslipidemia and pressure wave reflection still seems to be unclear, and it may be necessary to reconsider the clinical significance of vascular functional tests, particularly pressure wave reflection, in patients with dyslipidemia and to select patients who benefit most from the test.

Fourth, a bidirectional longitudinal association between the brachial-ankle PWV and radial AI was not observed in the present study, in contrast to the findings of their previous study [8]. This was explained by the distal shift of the pressure wave reflection point due to the underlying increased arterial stiffness in the subjects of the present study. Although the central BP and AI reflect the systemic arterial stiffness and tonus from large elastic arteries to peripheral small arterioles, there are currently no standard values for those tests. Thus, it is also necessary to standardize the measurement methods and determine their standard values. In addition, the combined evaluation of other physiological vascular functions, such as endothelial function, would be helpful to further assess vascular function and the severity of vascular failure [2]. Such a multidimensional approach will reveal the detailed manifestation of the vasculature and its severity of vascular failure, providing useful clinical information for risk stratification of cardiovascular events.

Fifth, readers may simply want to know the results of similar research in women because the characteristics of background cardiovascular risk factors often differ by sex. Age and sex are known to be major and fundamental determinants of vascular function. Indeed, various physiological functions, not limited to the vasculature, are expected to be more strongly affected by life events, including menopause, in women than in men during longitudinal observation periods. Because men are only approximately half of the population, it is crucial to elucidate sex differences in the longitudinal associations of cardiovascular risk factors with vascular function and to develop high-quality sex-specific cardiovascular medicine.

**Fig. 1:**
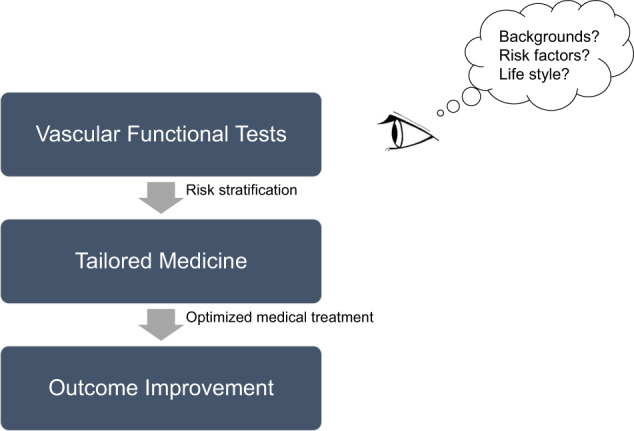
Preemptive medicine starting from vascular functional tests

In summary, given the study by Fujii et al. [4], it is necessary to optimize vascular functional tests according to individual medical backgrounds and situations and to perform tailored medicine according to those findings. Since vascular function and its values are affected by a variety of clinical factors, it is important to understand the function and its values comprehensively based on the underlying backgrounds. Thus, the most important goal is not to know the numerical value of vascular function but to stratify cardiovascular risks and individually optimize medical treatment through various clinical manifestations, including vascular function (Fig. 1). This integrated approach will surely contribute to an efficient reduction in the risk of cardiovascular events and improvement in outcomes, finally realizing preemptive medicine.
